# Artificial Intelligence Models Do Not Ground Negation, Humans Do. GuessWhat?! Dialogues as a Case Study

**DOI:** 10.3389/fdata.2021.736709

**Published:** 2022-01-24

**Authors:** Alberto Testoni, Claudio Greco, Raffaella Bernardi

**Affiliations:** ^1^Department of Information Engineering and Computer Science, University of Trento, Trento, Italy; ^2^Centre for Mind and Brain Sciences, University of Trento, Trento, Italy

**Keywords:** negation, multimodal models, transformers, multimodal encoders, visual dialogue, analysis

## Abstract

Negation is widely present in human communication, yet it is largely neglected in the research on conversational agents based on neural network architectures. Cognitive studies show that a supportive visual context makes the processing of negation easier. We take GuessWhat?!, a referential visually grounded guessing game, as test-bed and evaluate to which extent guessers based on pre-trained language models profit from negatively answered polar questions. Moreover, to get a better grasp of models' results, we select a controlled sample of games and run a crowdsourcing experiment with subjects. We evaluate models and humans against the same settings and use the comparison to better interpret the models' results. We show that while humans profit from negatively answered questions to solve the task, models struggle in grounding negation, and some of them barely use it; however, when the language signal is poorly informative, visual features help encoding the negative information. Finally, the experiments with human subjects put us in the position of comparing humans and models' predictions and get a grasp about which models make errors that are more human-like and as such more plausible.

## 1. Introduction

Negation is often neglected by computational studies of natural language understanding, in particular when using the successful neural network models. Very recently, a series of work have highlighted that negation is under-represented in existing natural language inference benchmarks (Hossain et al., 2020b) and that Pretrained Language Models have difficulty distinguishing a sentence from its negated form in fill-in-the-blank tests (Kassner and Schütze, 2020). This weakness of Language Models could have a strong impact on their success in real-life applications. For instance, Hossain et al. (2020a) show that the lack of a proper understanding of negation is an important source of error in machine translation and similarly, it would impact the quality of other applications based on natural language understanding, such as text summarization or personal assistants for health care or other uses. A recent contribution of AI to the society is the development of visual dialogue systems built on Pretrained Language Models. Clearly, they are an important tool for instance as personal assistants of visually impaired people (Gurari et al., 2018), but again their impressive achievements would be vanished if they fail to distinguish negative and affirmative information.

Admittedly, modeling negation is an ambitious goal, and even humans have a harder time understanding negative sentences than positive ones (Clark and Chase, 1972; Carpenter and Just, 1975). However, it has been shown that the presence of supportive context mitigates the processing cost of negation. In particular, this happens within dialogues (Dale and Duran, 2011), and when a visual context is given (Nordmeyer and Frank, 2014). Based on these findings, we argue that Visual Dialogues are a good starting point for making progress toward the ambitious but crucial goal of developing neural network models that can understand negation.

**Figure 1:**
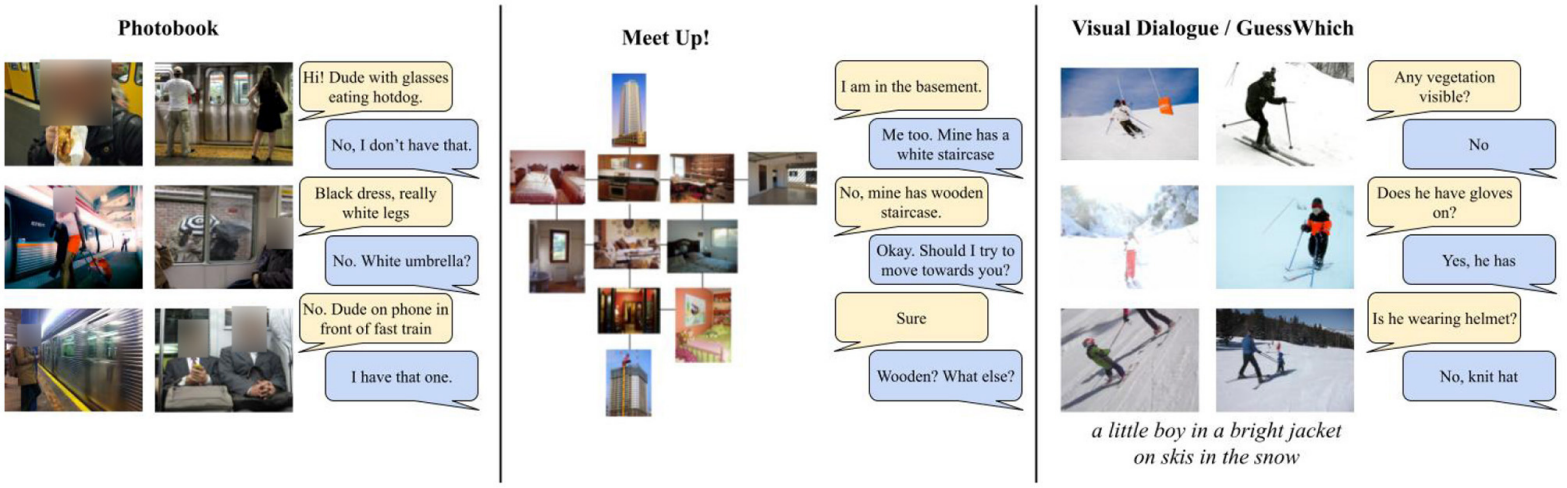
Examples of dialogues from two asymmetric and partially observable visual dialogue data [PhotoBook and Meet Up! (Haber et al., 2019; Ilinykh et al., 2019a)] and a symmetric visual dialogue in which the answerer sees the image and the questioner does not see it (Chattopadhyay et al., 2017; Das et al., 2017). For all datasets, we selected exchanges containing negation, the focus of our study.

**Figure 2:**
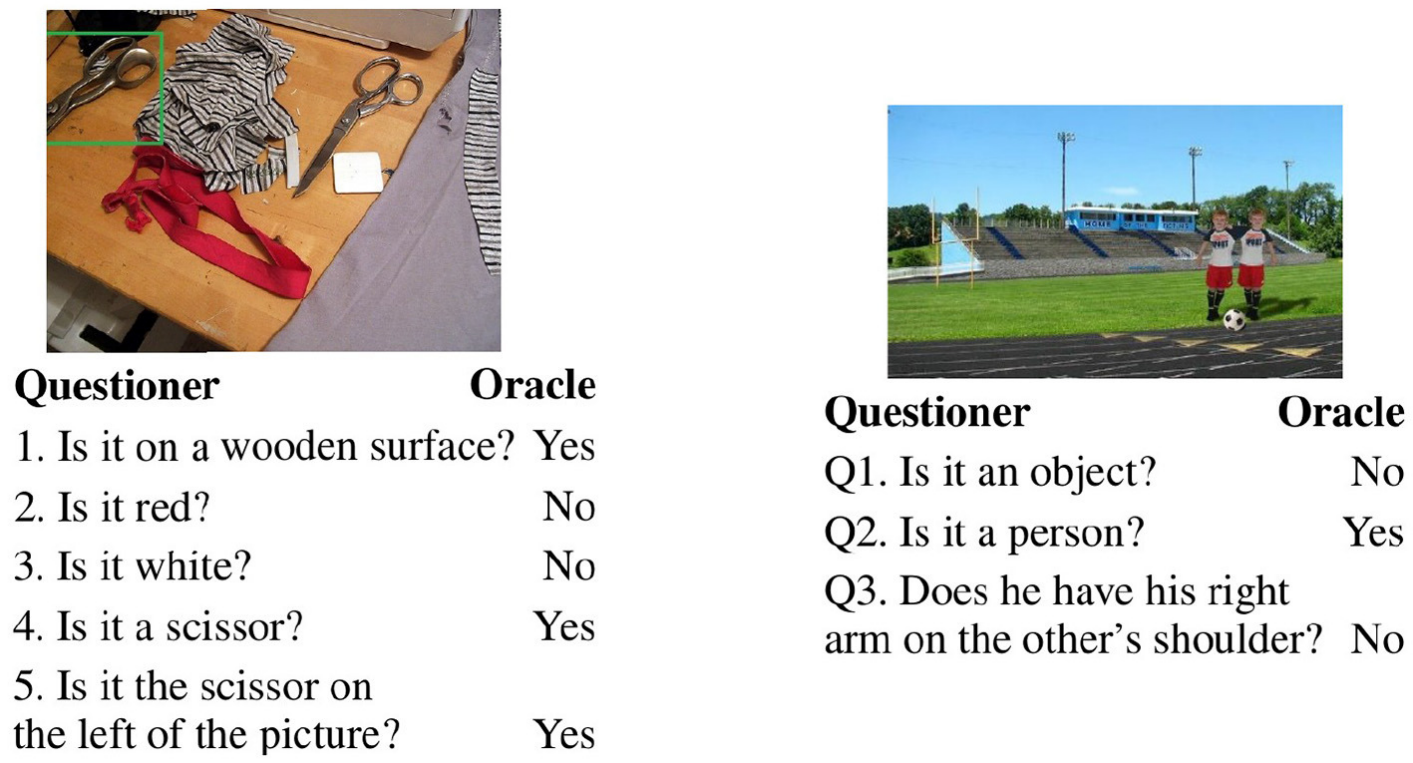
Two samples of GuessWhat?! human dialogues ending with a positive (left) and a negative (right) turn.

Visual Dialogues have a long tradition (e.g., Anderson et al., 1991). They can be chit-chat (e.g., Das et al., 2017) or task-oriented (e.g., de Vries et al., 2017; Haber et al., 2019; Ilinykh et al., 2019a,b). Task-oriented dialogues are easier to evaluate since their performance can be judged in terms of their task-success, hence we focus on this type of dialogues which can be further divided as following: the two agents can have access to the same visual information (de Vries et al., 2017), share only part of it Haber et al. (2019) and Ilinykh et al. (2019a) or only one agent has access to the image (Chattopadhyay et al., 2017). Moreover, dialogues can be symmetric (Haber et al., 2019), or asymetric, with one agent asking questions and the other answering it de Vries et al. (2017), Das et al. (2017), and Chattopadhyay et al. (2017). Finally, the dialogue turns can contain different speech acts (Haber et al., 2019; Ilinykh et al., 2019a,b) or only question anwer pairs (Chattopadhyay et al., 2017; Das et al., 2017; de Vries et al., 2017). The differences between the various type of dialogues are illustrated in Figure 1. As we can see symmetric games with partially observable data (PhotoBook and Meet up! Haber et al., 2019; Ilinykh et al., 2019a) sollicitate more complex exchanges than symmetric ones (Visual Dialogue, GuessWhich—the referentional game built from it Chattopadhyay et al., 2017; Das et al., 2017, and GuessWhat?! de Vries et al., 2017—the latter is illustrated in Figure 2). Given the difficulty negation poses to models, we take the scenario which is less complex from a dialogue perspective and in which questions are always grounded in the image: the one in which agents have access to the same visual information, only one agent can ask questions, and the questions are all of the same type. Hence, we take GuessWhat?! as case-study and focus on the referential grounded guessing task: a Guesser receives an asymmetric dialogue, consisting of Yes/No-questions over an image, a list of candidates and has to guess the target object the dialogue is about. In this setting, negation is heavily present as the answer to a binary question. As such it functions as a pointer to the alternative set of the negated expression; in other words it should be interpreted as pointing to the set of all the candidates objects which do not have the queried property.

GuessWhat?! dialogues have been collected by letting two humans play the game. As illustrated in Figure 2, such dialogues are quite simple: a sequence of rather short questions answered by “Yes” or “No” containing on average 30.1 (SD ± 17.6) tokens per dialogue. The dialogue length differs across the games since the questioner decides when he/she can stop asking questions and is ready to guess the target. To evaluate the extent models understand negatively answered questions, we take the human dialogues as input to the guesser. We select successful games, in other words those dialogues in which human players have succeeded in guessing the target object at the end of the game. We conjecture that within these dialogues a crucial role is played by the last turn whose role is to create a singleton alternative set and that this goal is achieved differently when the question is answered positively or negatively. In the former case, the question tends to almost fully describe the target object, whereas in the latter case it conclusively identifies the target object by excluding those candidates which most likely are not the target (Figure 2). To validate this conjecture, we run an online experiment with humans which set the ground for better evaluating the results obtained by models. We let humans and computational models perform the same task on the same controlled sample set. We compare encoders with respect to the architecture (Recurrent Neural Networks vs. Transformers), the input modalities (only language vs. language and vision) and the model background knowledge (trained from scratch vs. pre-trained and then fine-tuned on the downstream task). Our analysis shows that:

- While humans profit from negatively answered questions to solve the task, models struggle in grounding negation, and some of them barely use it;
- In No-turns, when the language signal is poorly informative, visual features help in processing the QA pair.

We hope that these results will stimulate more work on the processing of (grounded) negation and that the data we collected through our online experiment and its annotation will be a valuable contribution to such research direction.^1^

## 2. Related Work

### 2.1. Scrutinizing Visual Dialogue Encoding

Sankar et al. (2019) study how neural dialogue models encode the dialogue history when generating the next utterance. They show that neither recurrent nor transformer based architectures are sensitive to perturbations in the dialogue history and that Transformers are less sensitive than recurrent models to perturbations that scramble the conversational structure; furthermore, their findings suggest that models enhanced with attention mechanisms use more information from the dialogue history than their vanilla counterpart. We follow them in the choice of the architectures we compare, but we change the focus of the analysis by studying whether the polarity of the answer (Yes vs. No) affects the encoding of the information provided by the question-answer pair.

Kaushik and Lipton (2018) show that in many reading comprehension datasets, that presumably require the combination of both questions and passages to predict the correct answer, models can achieve quite a good accuracy by using only part of the information provided. Similarly to this work, we investigate how much models use the questions as well as the answers, provided by the Oracle, to select the target object among the possible candidates.

Interesting exploratory analysis has been carried out to understand Visual Question Answering (VQA) systems and highlight their strengths and weaknesses (e.g., Johnson et al., 2017; Kafle and Kanan, 2017; Shekhar et al., 2017; Suhr et al., 2017). Less is known about how well grounded conversational models encode the dialogue history and in particular, negatively answered questions. Greco et al. (2020) shows that pre-trained models transformers detect salient information in the dialogue history independently of the position in which it occurs. We build on their study to dive into how encoders represent positively vs. negatively answered questions within a visual dialogue.

### 2.2. SOTA LSTM-Based Models on GuessWhat?!

After the introduction of the supervised baseline model (de Vries et al., 2017), several models have been proposed. Zhao and Tresp (2018) has used attention mechanisms based on Memory Networks (Sukhbaatar et al., 2015) and (Shekhar et al., 2019) has proposed a model that is jointly trained to ask questions and guess the target. Building on the supervised learning step, all these models have been further trained with either some form of reinforcement learning (Zhang et al., 2018; Zhao and Tresp, 2018; Yang et al., 2019; Pang and Wang, 2020) or cooperative learning (Shekhar et al., 2019; Pang and Wang, 2020); this two-step process has been shown to reach higher task success than the supervised approach. Since our focus is on the Guesser and we are evaluating it on human dialogues, we will compare models that have undergone only the supervised training step.

### 2.3. Transformer-Based Models

The last years have seen the increasing popularity of transformer-based models pre-trained on several tasks to learn task-agnostic multimodal representations (Chen et al., 2019; Li et al., 2019, 2020; Lu et al., 2019; Tan and Bansal, 2019; Su et al., 2020). ViLBERT (Lu et al., 2019) has been recently extended by means of multi-task training involving 12 datasets which include GuessWhat?! (Lu et al., 2020) and has been fine-tuned to play the Answerer of VisDial (Murahari et al., 2019). Greco et al. (2020) have adapted the pre-trained transformer, LXMERT (Tan and Bansal, 2019), to the GuessWhat?! guessing task. Given the high accuracy achieved, we choose LXMERT as pre-trained transformer.

### 2.4. Visually Grounded Negation

Negation was already listed by Winograd among the linguistic phenomena a Grounded Conversational System should be able to interpret (Winograd, 1972). Significant progress has been obtained in the development of conversational systems based on neural network architecture; however, little is known about how these models interpret negation. Nordmeyer and Frank (2014) show that processing negation can be easier for humans if a visual context creates pragmatic expectations that motivate its use. However, it is unknown whether this holds for multimodal models. Suhr et al. (2019) show that SOTA models tested on visual reasoning often fail in properly grounding negative utterances. Gokhale et al. (2020) show that models have harder time in answering visual questions containing negation. Both studies look at negation as a logical operation, it reverses the truth value of the negated utterance. However, Oaksford (2002) show that humans often use negation not as a logical operator but rather as a way to create an alternative set of the negated expressions. This is exactly the role of the negative answer in the GuessWhat?! game. We are not aware of any study on Visual Dialogue that have tackled this issue.

## 3. Task and Dataset

In this paper, we run an in-depth analysis on how models integrate Yes/No answers into the question to solve the GuessWhat?! guessing task. We run a comparative analysis to evaluate the role of language priors and visual grounding, and we run a crowdsourcing experiment with subjects on a controlled sample of the games. Using a controlled sample set and knowing about humans' performance give us a better way to interpret the results obtained by the models on the full test set. Below we describe the task and training/validation set and the test sets we use through out the paper.

### 3.1. Task

GuessWhat?! (de Vries et al., 2017) is an asymmetrical game involving two participants who see a real-world image. One of the participants (the Oracle) is assigned a target object in the image and the other participant (the Questioner) has to guess it by asking Yes/No questions to the Oracle. de Vries et al. (2017) collected a human dialogue dataset via Amazon Mechanical Turk. Our focus is on multimodal encoding, hence we focus on the guessing task: given a human dialogue, consisting of Yes/No questions and their answers, an image and a list of possible candidate objects, the agent has to select the object the dialogue is about. Greco et al. (2020) have shown that human dialogue length is a good proxy of the guessing task difficulty^2^, where length is measured in terms of number of turns; for instance in Figure 2 the dialogue on the left is of length 5 (it consists of five turns) whereas the one on the right is of length 3. In the following, we use “turn” to refer to the position (of just the question or the answer or of the QA pair) within the dialogue.

### 3.2. Full Dataset

The GuessWhat?! dataset contains 155K English dialogues about approximately 66K different images from the MS-COCO dataset (Lin et al., 2014). We evaluate models using human dialogues, selecting only the games on which human players have succeeded finding the target and contain at most 10 turns (total number of dialogues used: 90K in training and around 18K both in validation and testing). Dialogues contain on average 4.5 Question-Answer (QA) pairs, the vocabulary consists of 4,901 words, and games have on average 8 candidates.^3^ The answer distribution is the following: 52.2% No, 45.6% Yes, and 2.2% N/A (not applicable). We divide the full test set into games whose dialogue ends in a Yes- vs. in a No-turn and obtain the Yes-set and No-set, whose statistics are reported in Table 1. As we can see, the two sets contain dialogues of the same average length, and similar number of candidate objects, hence their games are expected to be of similar difficulty. The last turns in these two subsets are expected to play a rather different role (as illustrated by the example in Figure 2): a Yes-question in the last turn is rather informative on its own, whereas a last turn answered negatively quite often needs the information gathered in the previous turns to be informative. On the other hand, we should note that last turns containing a negative answer are expected to be rather informative together with the dialogue history to guess the target. Hence, they are an interesting test-bed for our research question.

**Table 1:** Statistics on the full test set and on the Controlled test set; both divided into the Yes- (resp. No-) subsets obtained by selecting only dialogues with a positively (resp. negatively) answered question in the last turn.

	**Nr. Games**	**Av. Dialogue length**	**Av. nr candidates**
Full test set	18,840	4.5	8
Yes-set	16,366	4.5	8
No-set	2,350	4.5	7.8
Controlled sample	300	4.5	6.1
Yes-set	150	4.5	6.1
No-set	150	4.3	6.1

### 3.3. Controlled Sample

To compare models' results against humans' ones, we run an annotation experiment on a sample of games we carefully select. We consider dialogues consisting of 4- and 6-turns, and select those containing an equal number of Yes/No answers. Moreover, to control for the level of difficulty of the game, we select only games which have a maximum of 10 candidates. We obtain a subset with a balanced overall distribution of the two types of polar answers; it contains 1,491 games, of which 1,327 (resp. 164) contain in the last turn a question answered positively (resp. negatively). From these games, we randomly select 300 games (image, target) from the Yes- and No- test sets (150 each). In this way, we obtain a subset balanced also with respect of the polarity of the last question. We believe games in this sample set are equally difficult, considering the criteria discussed above.

## 4. Models

**Figure 3:**
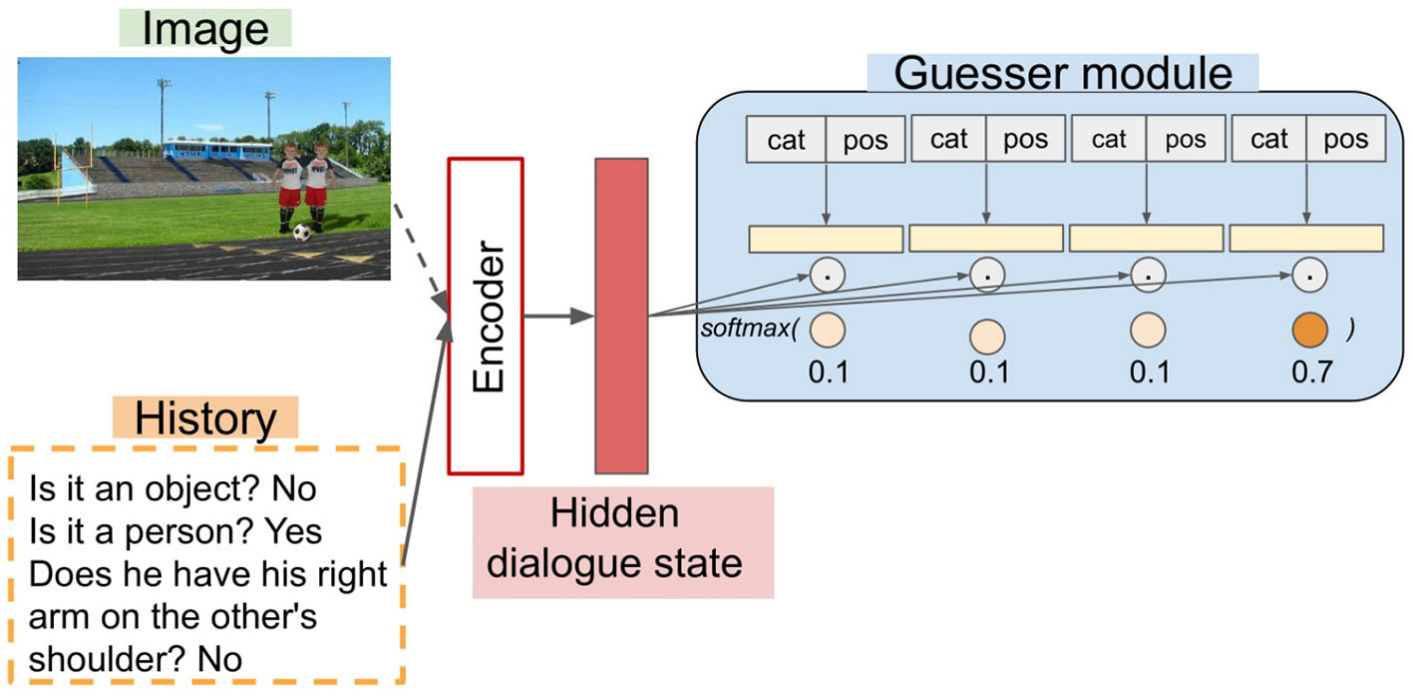
Shared Encoder-Guesser skeleton. The Guesser receives the category labels (e.g., “bottle”) and the spatial coordinates (pos) of each candidate object. Multimodal encoders receive both the image and the dialogue history, whereas blind models receive only the latter.

Following, Greco et al. (2020), all the guesser models we evaluate share the skeleton illustrated in Figure 3: an encoder paired with a Guesser module. For the latter, all models use the module proposed in de Vries et al. (2017). Candidate objects are represented by the embeddings obtained via a Multi-Layer Perceptron (MLP) starting from the category and spatial coordinates of each candidate object. The representations so obtained are used to compute dot products with the hidden dialogue state produced by an encoder. The scores of each candidate object are given to a softmax classifier to choose the object with the highest probability. The Guesser is trained in a supervised learning paradigm, receiving the complete human dialogue history at once. The models we compare differ in how the hidden dialogue state is computed. We compare LSTM vs. Transformers when receiving only the language input (Language-only, henceforth, Blind models) or both the language and the visual input (Multimodal, henceforth, MM models).

### 4.1. Language-Only Encoders

#### 4.1.1. LSTM

As in de Vries et al. (2017), the representations of the candidates are fused with the last hidden state obtained by an LSTM which processes only the dialogue history.

#### 4.1.2. RoBERTa

In the architecture of the model described above, we replace the LSTM with a robustly-optimized version of BERT (Devlin et al., 2019), RoBERTa, a SOTA universal transformer-based encoder introduced in Liu et al. (2019).^4^ We use RoBERTa_*BASE*_ which has been pre-trained on 160GB of English text trained for 500K steps to perform masked language modeling. RoBERTa was pretrained on several text corpora containing rather long utterances: BookCorpus (Zhu et al., 2015)+ English Wikipedia (as the original BERT model), CC-NEWS (Nagel, 2016), OPENWEBTEXT (Gokaslan and Cohen, 2019), and STORIES (Trinh and Le, 2018). It has 12 self-attention layers with 12 heads each. It uses three special tokens, namely CLS, which is taken to be the representation of the given sequence, SEP, which separates sequences, and EOS, which denotes the end of the input. We give the output corresponding to the CLS token to a linear layer and a *tanh* activation function to obtain the hidden state which is given to the Guesser. To study the impact of the pre-training phase, we have compared the publicly available pre-trained model, which we fine-tuned on GuessWhat?! (**RoBERTa**), against its counterpart trained from scratch only on the game (**RoBERTa-S**).

### 4.2. Multimodal Encoders

#### 4.2.1. V-LSTM

We enhance the LSTM model described above with the visual modality by concatenating the linguistic and visual representation and scaling its result with an MLP; the result is passed through a linear layer and a *tanh* activation function to obtain the hidden state which is used as input for the Guesser module. We use a frozen ResNet-152 pre-trained on ImageNet (He et al., 2016) to extract the visual vectors.

#### 4.2.2. LXMERT

To evaluate the performance of a universal multimodal encoder, we employ LXMERT (Learning Cross-Modality Encoder Representations from Transformers) (Tan and Bansal, 2019). It represents an image by the set of position-aware object embeddings for the 36 most salient regions detected by a Faster R-CNN and it processes the text input by position-aware randomly-initialized word embeddings. LXMERT is pre-trained on datasets containing rather short utterances: MSCOCO (Lin et al., 2014), Visual Genome (Krishna et al., 2017), VQA v2.0 (Antol et al., 2015), GQA balanced version (Hudson and Manning, 2019), and VG-QA (Zhu et al., 2016). Both the visual and linguistic representations are processed by a specialized transformer encoder based on self-attention layers; their outputs are then processed by a cross-modality encoder that, through a cross-attention mechanism, generates representations of the single modality (language and visual output) enhanced with the other modality as well as their joint representation (cross-modality output). Like RoBERTa, LXMERT uses the special tokens CLS and SEP. Differently from RoBERTa, LXMERT uses the special token SEP both to separate sequences and to denote the end of the textual input. LXMERT has been pre-trained on five tasks.^5^ It has 19 attention layers: 9 and 5 self-attention layers in the language and visual encoders, respectively and 5 cross-attention layers. We process the output corresponding to the CLS token as in RoBERTa. Similarly, we consider both the pre-trained version (**LXMERT**) and the one trained from scratch (**LXMERT-S**).^6^

## 5. Experiments on the Full Test set

We aim to understand whether models encode Yes/No answers and properly integrate them into the question. If answers play a role in the performance of the models in guessing the target object, removing them from the dialogues should cause a drop in the task accuracy. Following this conjecture, we evaluate models (at test time, without additional training) when receiving only the questions from the dialogues (without the answers). Moreover, as commented above, the last turn in the Yes-set vs. No-set is expected to play a rather different role. In particular, already alone a positively answered question in the last turn is expected to be rather informative whereas a last turn answered negatively is not. On the other hand, last turns containing a negative answer are expected to enrich the dialogue history and help to guess the target. Hence, in the following, we evaluate models aiming to understand the role of the last turn.

### 5.1. Accuracy Results

#### 5.1.1. Only Questions

We evaluate models when receiving dialogues containing only the questions.^7^ As expected, all models show an important drop as we can see from Table 2. Blind models have higher accuracy than the multimodal counterpart when receiving only the question, maybe because during training they learn to exploit the language surface more. Moreover, the pre-training phase helps to exploit the keywords in the questions as shown by the difference between the pre-trained and from scratch versions of both transformer based models. These results show that all models take the answers into account to some extent, and thus it is important to study their impact on the performance of the models.

**Table 2:** Full test set: Task Accuracy obtained by models when receiving: a) only the questions (Only Q); b) the full dialogue in the Yes-set vs. No-set, viz. games ending with a Yes-turn vs. a No-turn.

		**Full dialogue**	**Only Q**	**Full dialogue**	
		**all games**	**all games**	**Yes-set**	**No-set**
	Random	12.5	12.5	16.4	16.4
BLIND	LSTM	64.7	47.9	67.0	49.0
	RoBERTa-S	64.2	43.7	66.6	48.1
	RoBERTa	67.9	51.7	69.6	54.5
MM	V-LSTM	64.5	46.2	67.0	48.3
	LXMERT-S	64.4	32.0	66.6	49.5
	LXMERT	69.2	44.8	71.9	50.9

*All differences between RoBERTa and LXMERT are statistically significant*.

#### 5.1.2. Dialogues With a Yes- vs. No- Answer in the Last Turn

We now investigate how the polarity of the final answer in the dialogue affects the performance in the guessing task. Models reach a rather lower accuracy on the No-set, suggesting that models have harder time interpreting dialogues ending with a negative answer (Table 2). Differently from what one would expect, it seems the pre-trained transformer that does not have access to the visual representation of the “alternative set” (RoBERTa) performs better than the multimodal model, LXMERT, in the challenging No-set games. It is not clear, however, where the advantage of RoBERTa comes from. Hence, in the next section, we aim to understand these results better by using the controlled sample and comparing models against the humans' performance, with a particular focus on the role of the last dialogue turn.

#### 5.1.3. The Role of the Last Turn

To analyse the role of the last turn, we compute models' accuracy when receiving the dialogues without the last turn or with only the last turn. The drop obtained from the setting in which models have access to the full dialogue quantifies the role of the last turn. First of all, as shown in Table 3, when removing the last turn in the Yes-set, LXMERT has a higher drop in accuracy than RoBERTa: −22.0% (from 71.9 to 49.9) vs. –16.1% (from 69.6 to 53.5); the fact that LXMERT relies on the last turn a lot might be due to LXMERT having harder time than RoBERTa in encoding the dialogue history, as observed in Greco et al. (2020). When only the last turn is provided, LXMERT profits from the pre-training phase more than RoBERTa. Recall that LXMERT has seen shorter text than RoBERTa during training, e.g., MS-COCO captions vs. Wikipedia text. This difference could be behind such results. In the No-set, LXMERT processes the last turn better than RoBERTa (it reaches 26.6 accuracy when receiving only the last turn, +3.3 than RoBERTa), but again it has more difficulty in integrating such information with that gathered through the dialogue history (it scores –3.4% than RoBERTa when receiving the full dialogue). Finally, as expected, when receiving only the last turn, models obtain a high accuracy when the answer is positive (Yes-set) and are near to chance level when it is negative (No-set). Interestingly, in the No-set, RoBERTa and LXMERT have a rather similar accuracy when the last turn is not given and LXMERT does slightly better than the language encoder when receiving only the last turn. These results suggest that the advantage of RoBERTa over LXMERT highlighted in Table 2 is due to a better processing of the whole dialogue history, while LXMERT exploits better shorter sequences such as the last turn taken individually in the No-Set (Table 3).

**Table 3:** Full test set: Accuracy comparison when giving to the model the dialogue without the last turn (W/o Last) or with only the last turn (Last).

	**Yes-set**		**No-set**	
	**W/o Last**	**Last**	**W/o Last**	**Last**
LSTM	48.3	51.8	39.9	24.5
RoBERTa-S	49.8	50.7	39.6	21.8
RoBERTa	53.5	55.6	42.5^*^	23.3
V-LSTM	48.6	47.3	37.8	20.7
LXMERT-S	48.4	51.7	41.0	22.2
LXMERT	49.9	61.2	41.9^*^	26.6

* *(The marks RoBERTa's and LXMERT's scores whose differences are statistically not significant)*.

#### 5.1.4. Tests of Statistical Significance

To validate our findings about the comparison between RoBERTa and LXMERT, we have run the McNemar's test with a significance level of 0.05. We use an asterisk to signal scores whose differences is not significant (Tables 2, 3).

### 5.2. Guesser's Probability Distribution

We now analyze how the guesser module assigns probabilities to the target object across the turns to understand better the role of positive and negative answers at a more fine-grained level. We compute how the probability assigned by the Guesser to the target object *P*(*o*) changes after each turn (*P*(*o*)_*T*_*i*+*i*__−*P*(*o*)_*T*_*i*__) and compare turns *T*_*i*_ with a Yes, No or N/A answer. We expect it is easier to use the Yes-turns than the No ones, but we hope models are able to benefit from the questions answered negatively more than those answered by N/A. Moreover, we focus on the games in which the Guesser succeeds to select the target object, and quantify the effect of the last turn on the probability assigned to the target. We expect the change in the last turn of the No-set to be much higher than No-turns in average, whereas this should not happen with last turn in the Yes-set.

Although the average probability assigned to the target is similar before a Yes-turn and a No-tun for all models,^8^ questions answered with Yes bring a much higher increase of probability than questions answered with No—which for LSTM have on average the same impact as those answered by N/A (2.9 vs. 2.3) (Table 4).^9^ Again, RoBERTa is the model that seems to profit of the negative turn more: the probability the guesser assigns to the target object after a No-turn increases of 5.9 vs. 4.1 when using LXMERT as encoder. However, when we focus on the last turn (Table 4-right), LXMERT is the model for which the negative answer brings a higher increase to the target object In the following, by zooming into the controlled sample we aim to get a more accurate comparison of models with respect to the specific issue of how they encode negatively answered questions.

**Table 4:** Change across consecutive turns in the probability assigned to the target after Yes- vs. No- vs. N/A-turns, i.e., *P*(*o*)_*T*_*i*+1__−*P*(*o*)_*T*_*i*__ (full dialogue history in the full test set) and before/after the last turn (Last turn in games on which the model has succeeded).

	**All games**			**All successful games**		
	**Full dialogue history**			**Last turn**		
	***T*** **_*i*_:*Yes***	***T*** **_*i*_:*No***	***T*** **_*i*_:*N*/*A***	***T*** **_*i*_:*Yes***	***T*** **_*i*_:*No***	***T*** **_*i*_:*N*/*A***
LSTM	14.5	2.9	2.3	26.3	16.2	6.3
RoBERTa-S	12.7	3.5	1.9	24.6	16.4	1.1
RoBERTa	12.3	5.9	1.4	22.9	18.8	1.1
V-LSTM	14.0	3.1	2.9	23.7	13.7	6.7
LXMERT-S	12.3	4.4	2.1	24.8	19.3	0.7
LXMERT	16.4	4.1	1.4	30.0	24.9	3.2

### 5.3. Summary

In short, the experiments run so far show that all models take the answer of the asymmetric GuessWhat?! dialogues into account. The pre-trained encoders are the best models over all games and are on par with one another in processing positively answered questions. But, the results on the Yes-set when removing the last turn or when giving only the last turn shows that LXMERT profits from the last Yes-turn more than RoBERTa. We conjecture this is due to the fact that LXMERT has a harder time encoding the dialogue history. The overall accuracy obtained on the No-set suggests that RoBERTa encodes the negatively answered questions better than LXMERT. However, an in-depth analysis of the Guesser probability distribution shows that the Guesser profits from the last turn in the No-set more when it is based on LXMERT than on RoBERTa. From the analyses presented so far, it emerges that the models we considered have different strengths and weaknesses, depending on many factors. To establish an upper-bound for models' performance and to assess the severity of the errors made by the models, in the following we present an in-depth analysis we carried out with human annotators playing the same guessing task of the models.

## 6. Controlled Sample: Humans and Models

In order to interpret models' performance on encoding Yes/No-turns, we evaluated humans' performance on the controlled games sample described in section 3. These results set an upper-bound for model performance, and give us a powerful tool to better scrutinize our results.

### 6.1. Experiments and Results With Human Annotators

We asked human annotators to perform the GuessWhat?! guessing task on a controlled sample of test set games. Similarly to what discussed in section 5, we evaluate several settings: we provide annotators with the full dialogue, the dialogue without the last turns, or only the last turn. Moreover, to check the average informativeness of Yes- No-turns, we add the setting in which we remove from the dialogues all turns of the same polarity.

#### 6.1.1. Data Collection

Through Prolific,^10^ we collected complete annotations from 48 subjects who were paid Euro 8.27/h. Each participant annotated 75 games from one of the four settings. In total, we have collected 3600 human answers. Each setting has received annotation from 3 participants. Participants were asked to be native English speakers.

**Figure 4:**
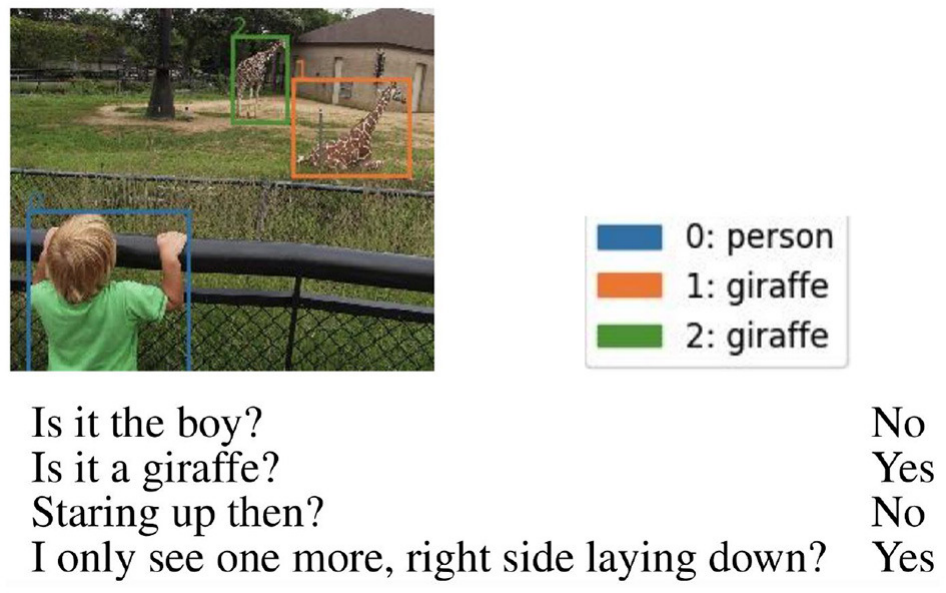
Prolific interface: Humans were given a dialogue, an image with colored bounding boxes, and a numbered list of candidates with colors matching those of the bounding boxes. They had to use the keyboard device to choose the target.

Participants were given an image with bounding boxes associated with each candidate object, together with a progressive ID number, as illustrated in Figure 4. They express their guess by pressing on the device's keyboard the number corresponding to the chosen object. Before starting the experiment, they were shown three trial games for which the correct answer was displayed in case the annotator chose the wrong target. We added two control games in each setting, i.e., games with a full dialogue history and few candidate objects. Participants were told there where control games and that they would have been excluded from the data collection in case the wrong answer was given for those games. Only one annotator wrongly guessed the control games and was therefore excluded. We recorded the time taken by each participant to complete the experiment. On average, humans took 12.23 s for each datapoint in the group A (removing turns), 15.55 s for group B (without last turn), 10.52 s for group C (only last turn), and finally 20.26 for group D (full dialogue). We found no statistically significant correlation between the time taken to guess the target and the success in solving the task.

#### 6.1.2. Tests of Statistical Significance

As we did in the previous section, we validate the accuracy results by running a McNemar's test with a significance level of 0.05 (Tables 5, 7). Table 6 reports the times taken by humans to play games belonging to the different groups we have analyzed. The differences within groups are not normally distributed—Shapiro–Wilk test. Hence, to check the validity of such comparisons we have run a Wilcoxon rank-sum statistic for two samples using 0.05 as significance level. Again, we use asterisks to signal the results whose difference is not statistically significant.

**Table 5:** Humans' performance on controlled sample: percentage of games guessed correctly by at least two participants (MAJ) vs. by at least one participant (MIN).

	**A) Removing turns**			
MAJ	Only Yes	66.00		
	Only No	46.00		
MIN	Only Yes	80.67		
	Only No	72.67		
		**B) W/o last**	**C) Only last**	**D) Full dialogue**
MAJ	Yes-set	75.33	71.33	86.67^*^
	No-set	49.33	30.67	80.67^*^
MIN	Yes-set	92.00	88.00	98.00
	No-set	64.67	58.00	90.00

* *Not significant*.

**Table 6:** Average time (seconds) taken by humans to solve games belonging to the different groups analyzed.

**Group**	**Description**	**Average time/token (s)**
A	only yes turns	0.45
A	only no turns	0.57
B	without last (yes)	0.94^*^
B	without last (no)	0.79^*^
C	only last (yes)	1.20
C	only last (no)	2.53
D	full dial ending with yes	0.72
D	full dial ending with no	0.85

*Normalized with respect to the number of token in the text; only successful games are considered*.

* *not significant*.

#### 6.1.3. Results With Humans

As mentioned above, we focus on games on which human players have been successful in guessing the target object. It has to be noted that during the GuessWhat?! data collection, each game was played only once and the target object was guessed by the same player who asked the questions. Hence we do not know whether the same dialogue-image would be equally informative for another player to succeed in the game neither we know the level of uncertainty behind the choice made by the successful player. With these questions in mind, in Table 5 we report the accuracy obtained by humans in our controlled experiment by considering a game successfully solved if (a) at least one participant correctly identifies the target object among the list of candidates (the typical GuessWhat?! accuracy evaluation setting, modulo the fact that in our case the questions are already asked) and (b) at least two participants guess the target correctly (the most standard and solid evaluation); we refer to these two accuracy metrics as minority (MIN) and majority (MAJ) schema, respectively.

Given that we are working with games on which GuessWhat?! human players succeed guessing the target, the fact we do not obtain 100% accuracy in the group D (complete dialogues) is by itself interesting. The difference between the two schema shows that, also in the games successfully solved by human players in the GuessWhat?! dataset, there is a margin of uncertainty. As we see from the Table 5 (Group D, full dialogue), 98.00% of the games ending with a Yes-turn could be guessed by at least one participant (minority schema) whereas 86.67% of them were guessed correctly by at least two participants (majority schema). Games ending with a No-turn are more difficult: 90.00% (resp. 80.67%) of the games could be guessed based on the minority (resp. majority) schema. However, whereas the difference between the Yes- vs. No-set in the minority schema is significant it is not so in the majority schema. This suggests that, for humans, the level of difficulty of the two subsets is similar. The results on Group A (removing turns) shows that on average Yes-turns are more informative than No-turns. As expected, the last turn in the Yes-set is quite informative: with only the last turn (Group C), humans' accuracy drops of only –10.00% (resp. –15.34) reaching 88.00 (resp. 71.33) accuracy in the minority (resp. majority) schema. Furthermore, the last turn in the Yes-set is quite redundant with the information provided by the previous turns: when receiving the dialogue without the last turns (Group B), humans' accuracy drops of only 6% (resp. 11.34) in the minority (resp. majority) schema. Instead, the last turn in the No-set seems to provide further information that needs to be integrated with those received in the previous turns: without the last turn the accuracy on the No-set drops of 25.33 (resp. 31.34). All in all, these results show that also for humans gathering information from the No-turn is harder than with the Yes-turn, yet the last turn in the No-set is informative and humans manage to profit from it to succeed in the task relatively well. This result highlights the value of negation in visual dialogues, and show why it is an important requirement for computational models to properly process it.

To measure the processing cost of negative turns, we have analyzed the average time taken by human to correctly solve games belonging to the four categories we discussed so far. Table 6 shows that interpreting questions answered positively is faster than interpreting the ones answered negatively, and this result holds for all settings. In particular, processing positively-answered questions takes less than processing negatively-answered ones (group A), and a final positive turn is processed much faster than a negative final turn (group C). Interestingly, in the Yes-set guessing the target is faster when receiving the full dialogue than when receiving the dialogue without the last turn (0.72 vs. 0.94 s/token, *p* < 0.05), this might be due to what observed above, namely the last Yes-turn summarizes the salient information collected till that point and hence speeds up the choice. Whereas the negative answer in the last turn brings a boost in performance, it does not affect significantly the time taken by human annotators to process the dialogue (0.79 vs. 0.85 s/token, *p* > 0.05). These results show that the time taken by human participants to solve the game mirrors the processing cost of negation, which is also influenced by the context (dialogue) in which it appears.

#### 6.1.4. Results Humans vs. Models

We now evaluate the models on the same controlled sample of games we used with human annotators. In Table 7, we report the task accuracy obtained by models when removing all the Yes turns (remaining with only No-turns) or all the No-turns (remaining with only Yes-turns). As can be seen from the table, the performance of the two best models is rather similar: both in the full dialogue and in the only Yes-turns the difference between their results is not significant. Similarly to humans, models accuracy drops less when receiving only the Yes-turns than when receiving only the No-turns. However, models' overall accuracy when receiving the full dialogue is far from the human upper-bound even when using the majority vote schema. As we can see in Table 8 this rather big difference between models and humans is due to the No-set: while humans correctly succeed in 80.67% of the games ending in a No-turn, models reach at most the 50%. It is thus clear that if it is true that negation has a higher processing cost for both humans and computational models, the latter struggle to profit from negatively answered questions.

**Table 7:** Controlled sample.

		**Full dialogue**	**Removing turns**	
			**Only No-turns**	**Only Yes-turns**
**BLIND**	**Random**	**16.5**	**16.5**	**16.5**
	**LSTM**	**57.0**	**30.67**	**48.00**
	**RoBERTa-S**	**54.66**	**29.33**	**50.00**
	**RoBERTa**	60.0^*^	**35.33**	52.00^**^
**MM**	**V-LSTM**	**55.66**	**25.33**	**50.66**
	**LXMERT-S**	**54.33**	**32.66**	**48.00**
	**LXMERT**	59.67^*^	**25.33**	56.66^**^
	**Human (MAJ)**	**83.67**	**46.00**	**66.00**

*Removing turns: comparison of the task accuracy when models receive the full dialogue vs. only the No- vs. only the Yes-turns. Human accuracy computed with the majority vote*.

* *,*

** *not significant*.

**Table 8:** Controlled sample.

		**Yes-set**			**No-set**		
		**Full**	**W/o**	**Only**	**Full**	**W/o**	**Only**
		**dialogue**	**last**	**last**	**dialogue**	**last**	**last**
BLIND	LSTM	68.00	55.30	51.30	46.00	34.00	30.00
	RoBERTa-S	64.67	49.33	48.67	44.67	39.33	27.33
	RoBERTa	71.33	55.33	63.33	48.67	40.67	22.00
MM	V-LSTM	60.67	49.33	49.33	50.67	34.67	16.67
	LXMERT-S	61.33	50.00	47.33	47.33	36.00	22.00
	LXMERT	71.33	53.33	60.67	48.00	46.00	31.33
	Humans (MAJ)	86.67	75.33	71.33	80.67	49.33	30.67

*Without the last turn, Only the last turn, Full Dialogue: accuracy comparison to highlight the role of the last turn when it contains a positive (Yes-set) vs. negative (No-set) answer*.

### 6.2. Comparison With Humans' Errors

In the following, we run an error analysis by comparing models and humans on their failures. We expect that a model that properly grounds the dialogues is likely to make human-like mistakes. To this end, among the games failed by a model, we check how many of them have been failed by at least one human annotator (Table 9); moreover, in the games in which a model and at least one participant failed, we check whether the error made by the model and the participant is exactly the same, i.e., if they have chosen the same (wrong) candidate object (Table 10). As we can see from Table 9, LXMERT is the model whose failed games are most similar to the ones failed by human annotators. However, if we look (in a more fine-grained way) at the exact candidate objects they select, we found that RoBERTa is the model whose errors are more human-like for most of the settings (Table 10). This analysis highlights how human annotations help interpret models' results and evaluate the quality of their predictions.

**Table 9:** Error Analysis: Percentage of games human failed among those failed by each model.

	**Removing turns**	**W/o last**	**Only last**	**Full dialogue**
V-LSTM	80.65	73.56	78.61	53.38
LXMERT-S	82.12	71.35	81.12	59.12
LXMERT	83.05	77.48	85.19	58.68
RoBERTa-S	82.87	73.65	80.65	55.88
RoBERTa	83.43	72.44	82.56	55.00

**Table 10:** Error Analysis: Percentage of games in which each model does the same mistake made by humans (i.e., by selecting the same wrong candidate object as a human annotator).

	**Removing turns**	**W/o last**	**Only last**	**Full dialogue**
V-LSTM	45.33	48.44	41.14	49.30
LXMERT-S	52.38	52.46	42.77	51.85
LXMERT	51.70	58.12	47.10	49.30
RoBERTa-S	57.33	51.22	46.67	44.74
RoBERTa	60.99	51.33	53.52	53.03

**Figure 5:**
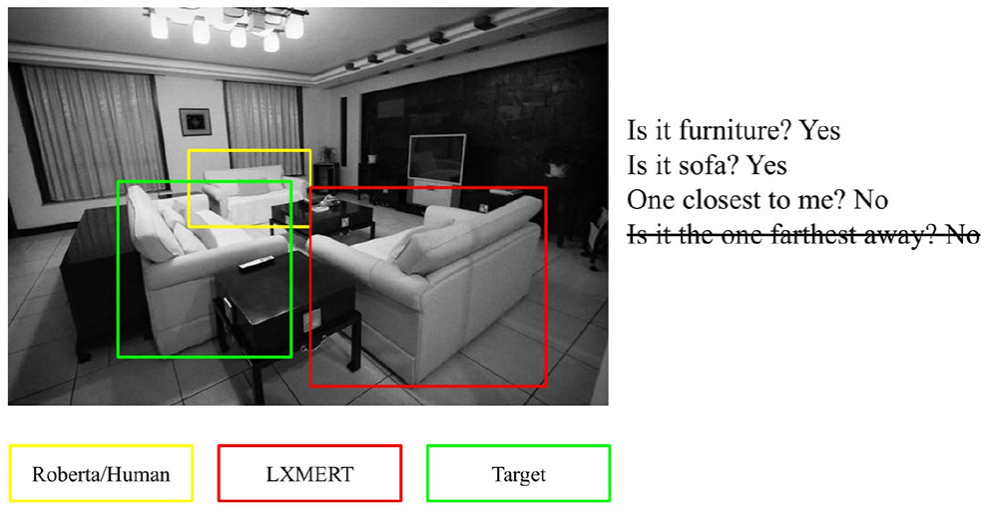
Errors made by humans and computational models when receiving dialogues without the last turn.

In Figure 5, we report a game in which both models and humans failed to guess the target when the last turn was not given; interestingly, at that stage, with only the first three turns, the selection made by RoBERTa and humans could be valid. This shows that checking when models and humans make the same mistakes gives a hint about which errors are plausible. From our qualitative analysis, it seems that RoBERTa takes spatial questions into account more than LXMERT, maybe because it exploits the spatial coordinates of the candidate objects whereas LXMERT overrides that information with the one it receives from the visual features. More in-depth analysis is required to assess what factors most influence the outcome of the models.

### 6.3. Summary

The evaluation of models on the controlled sample confirms that RoBERTa and LXMERT behave rather similarly on the Yes-set across all settings. More interestingly, it shows that in the No-set LXMERT is closer to humans than RoBERTa considering the accuracy in the task. LXMERT seems to be failing in the integration of the last No-turn with the dialogue history: its accuracy is similar to humans in the settings without last and only last turn, but it is far from them when the whole dialogue is given. Moreover, visual features seem to be of more help in the No-set than in the Yes-set: in the Yes-set across the controlled groups, the blind models do better or similar to their multimodal counterpart, whereas on the No-set the opposite holds. Finally, our error analysis reveals that RoBERTa is the model whose predictions are most human-like when it fails to identify the target object.

## 7. Discussion and Conclusion

In the current AI research, driven by the success of language models and neural network architectures, negation is under-studied. Dialogue history and visual context have been shown to facilitate the processing of negation in humans. Hence, we took negation in visual dialogues as our object of investigation and studied how SOTA multimodal models profit from negatively answered questions (No-turns) in the GuessWhat?! game. Such negative information is informative for humans to succeed in the game, and this holds in particular when the No-turn occurs as the last one of a game in which the human player has been successful in guessing the target. Therefore, we focus attention on the subset of dialogues ending with a No-turn and compare them with those ending with a Yes-turn. Our results show that SOTA models' performances on these two sub-sets is rather different, e.g., LXMERT obtains 71.9 vs. 50.9% accuracy in the Yes- vs. No-set, respectively (Table 2). To better interpret these results, we have run an online experiment with humans: we carefully selected a controlled sample of games and asked subjects to play the role of the guesser. We evaluated models' behavior on such a controlled sample of games and used humans' results to better interpret the success and failures of models. The analysis shows that humans are much faster in processing positively answered questions than negatively answered ones. Yet, they do profit from the latter to succeed in the referential guessing task reaching 80.67% accuracy in the No-set – on which models guess correctly barely the 50% (Table 8). This shows that models are far away from the human ability to ground negation and we believe efforts should be put to reduce this important gap between humans and models' performance.

Our findings can help design models which could ground negation better than current SOTA models. First of all, our comparison between the accuracy obtained by LXMERT and RoBERTa in the various settings (Tables 2, 3, 8) suggests that LXMERT grounding of negation within a dialogue could be improved by pre-training it on longer text. One could consider adding task-oriented dialogues in the pre-training phase Moreover, our comparison of models' and humans' errors leads us to conjecture that LXMERT fails to exploit the spatial information provided in the dialogue, this could be behind the fact that though it grounds negation in short texts better than RoBERTa, the latter's mistakes are more human-like, since humans rely on such information to locate and identify the target object. This limitation of the LXMERT based Guesser could be overcome by building a model that exploits the image regions received as input to perform the task, similarly to what has been recently proposed in Tu et al. (2021) for another multimodal model. Finally, Hosseini et al. (2021) shows that pre-trained language models can better understand negation if trained with an unlikelihood objective. This is a first important step ahead in modeling negation in the neural network-era, but the model's performance on entailment judgments involving negation is still low. Cognitive sciences findings on human processing of negation show that humans profit from expectations driven by the visual context to process negative information quickly and effectively Nordmeyer and Frank (2014); we believe that models should be trained to exploit more such expecations and that a (multimodal) communicative setting can help bring a boost for learning to encode (grounded) negation.

The results we obtain do not always provide conclusive answers, but we believe they convincingly show the weakness of current multimodal encoders in processing negation and represent a starting point toward future research. We started from the observation that dialogue history and visual context makes the processing of negation easier for humans. To fully understand whether this can be the case for models too, a comparison on processing negation in language-only vs. multimodal settings should be carried out. To this end, the study could be extended to other dasests in which the visual input is not shared or only partially shared by the agents, such as VisDial, PhotoBook and Meet up! (Haber et al., 2019; Ilinykh et al., 2019a) or language-only task-oriented dialogues (e.g., those used in Wu et al., 2020). Moreover, negative information can be conveyed in different ways, but we have studied only the easiest: a straightforward negative answer to a binary question. It would be interesting to explore the use of negation in declarative sentences and in more complex interactions. Finally, though our study builds on observations about the information gain the guesser accumulates through the dialogue at each turn, we have taken the dialogues as static blocks. A study about how humans and models incrementally gain information through the dialogue should be run to better understand their behavior.

To conclude, our findings have theoretical and also practical implications: for humans, negatively answered questions can be as informative as affirmatively answered ones; a system that is not able to properly handle negation may be detrimental in real-world scenarios. More research should be done on the issue to better understand whether neural network architectures can learn to ground negation on the alternative set it activates. To this end, we might need to single out various issues that are entangled in our analysis. First of all, it would be beneficial to have a multimodal dataset designed for this purpose. Secondly, when evaluating universal encoders the difference in the pre-training data is a confounder that should be avoided. Finally, it would be useful to have a large-scale human behavioral experiment that takes into account the incremental information gain at the core of a task-oriented dialogue exchange. We believe such data to be crucial both for training models to properly ground negation and for evaluating not only their task success but also their inside mechanisms as advocated for instance by Zhang et al. (2019). Once models learn to encode negation in grounded contexts, the next step will be to transfer such skills to language-only settings by exploiting transfer learning methods (e.g., Ruder, 2019).

## Data Availability Statement

The raw data supporting the conclusions of this article will be made available by the authors, without undue reservation.

## Ethics Statement

Ethical review and approval was not required for the study on human participants in accordance with the local legislation and institutional requirements. The patients/participants provided their written informed consent to participate in this study.

## Author Contributions

AT: design of computational experiments, model implementation and evaluation (Sections 5.1 and 5.2), human data collection and analysis (Section 6), and writing. CG: design of computational experiments, model implementation, and evaluation (Section 5.1), Supplementary Material, and writing. RB: research question, design of computational experiments, human data collection, and analysis (Section 6), writing. All authors contributed to the article and approved the submitted version.

## Conflict of Interest

The authors declare that the research was conducted in the absence of any commercial or financial relationships that could be construed as a potential conflict of interest.

## Publisher's Note

All claims expressed in this article are solely those of the authors and do not necessarily represent those of their affiliated organizations, or those of the publisher, the editors and the reviewers. Any product that may be evaluated in this article, or claim that may be made by its manufacturer, is not guaranteed or endorsed by the publisher.

## Footnotes

^1^ https://github.com/albertotestoni/annotation_human_gw

^2^In shorter dialogues the area of the target object is bigger than in longer dialogues, and in short dialogues the target object is quite often a “person” – the most common target category in the dataset; moreover, the number of distractors in longer dialogues is much higher.

^3^The dataset of human dialogues is available at https://guesswhat.ai/download.

^4^We have also tried BERT, but we obtained higher accuracy with RoBERTa.

^5^Masked cross-modality language modeling, masked object prediction via RoI-feature regression, masked object prediction via detected-label classification, cross-modality matching, and image question answering.

^6^We use the code available from https://github.com/claudiogreco/aixia2021.

^7^We replaced all the answers with the “unknown” [UNK] token.

^8^The difference is lower than 10%.

^9^In Table 4 we report the results for all the dialogues. Similar patterns have been seen when comparing the models on games with a given number of candidate objects.

^10^ https://www.prolific.co/
