# Supplementary material for: Artificial Intelligence Models Do Not Ground Negation, Humans Do. GuessWhat?! Dialogues as a Case Study
Source: Front Big Data. 2022 Jan 24;4:736709. doi: 10.3389/fdata.2021.736709 (PMC8819179; doi:10.3389/fdata.2021.736709)
Supplement: Supplementary file 1 [file Data_Sheet_1.PDF]

Artificial Intelligence models do not ground negation, humans do. GuessWhat?! dialogues as a case study Supplementary Material

Alberto Testoni [1*†], **Claudio Greco** [2†] **and Raffaella Bernardi** [1,2]

[1] *DISI, University of Trento, Trento, Italy*
[2] *CIMeC, University of Trento, Trento, Italy*

Correspondence*:
Alberto Testoni, Via Sommarive 9, I 38123, Povo (TN), Italy
alberto.testoni@unitn.it

1 GAME EXAMPLES

It seems that RoBERTa takes spatial questions into account more than LXMERT, maybe because it exploits the spatial coordinates of the candidate objects whereas LXMERT overrides that information with the one it receives from the visual features. An example of dialogue where RoBERTa shows its strength on spatial questions is shown in figure 1. In this dialogue, models receive only the last turn and RoBERTa successfully exploits the spatial information of the last turn to correctly guess the fork which is the closest candidate to the camera, whereas LXMERT selects the wrong target.

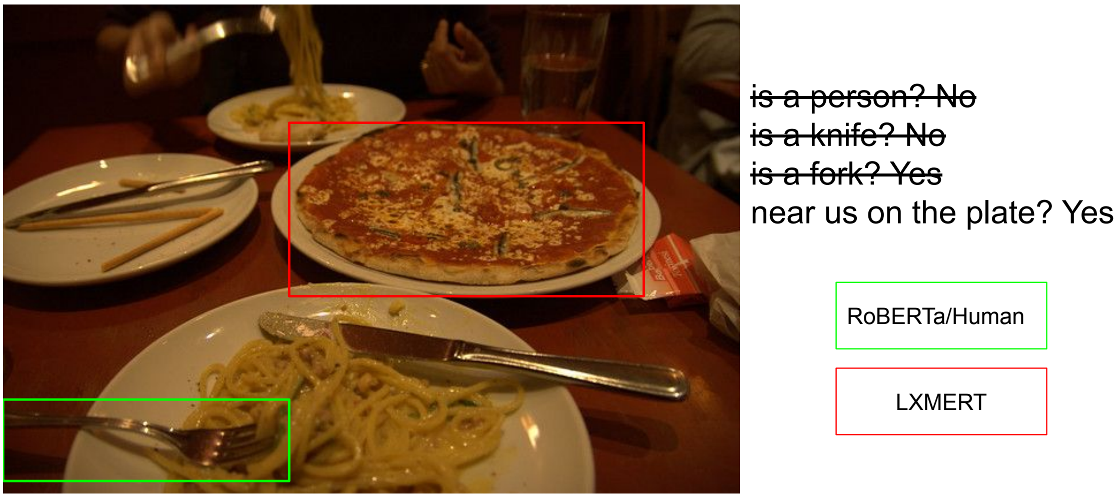

Figure 1. Example of game where RoBERTa correctly guesses the object, but LXMERT does not.

LXMERT seems, instead, to shine when grounding questions which involve to recognize objects in the image. For instance, figure 2 shows an example of game where LXMERT correctly guesses the baby on the right, but RoBERTa does not.

† These authors have contributed equally to this work.

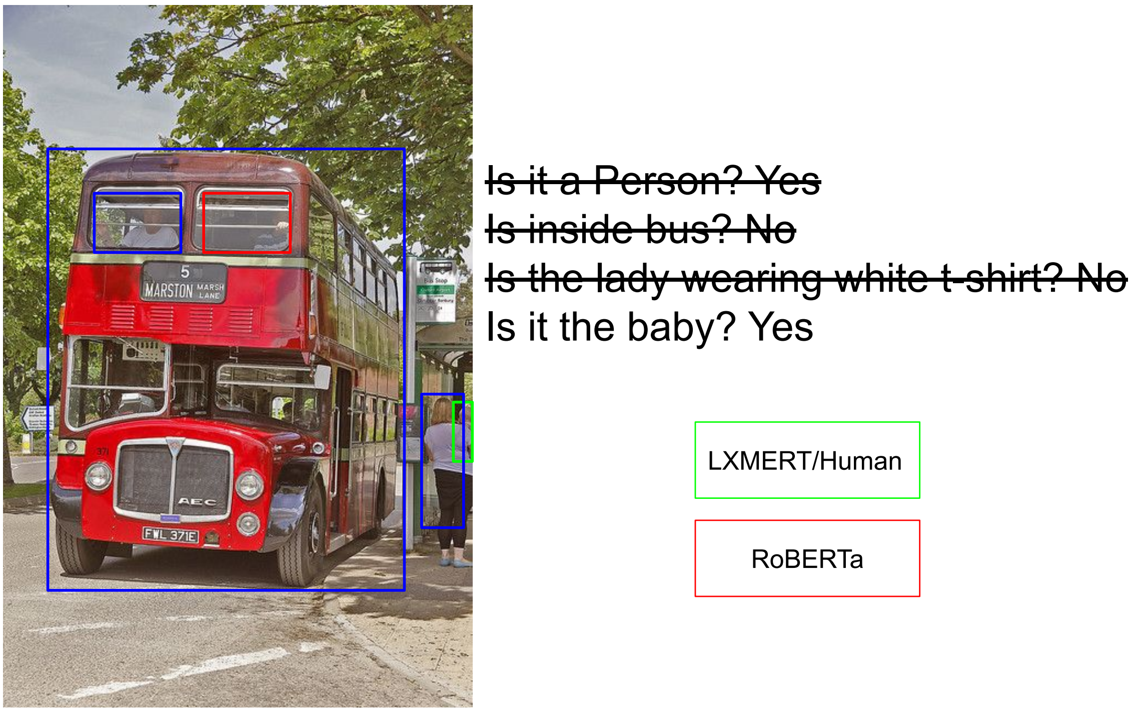

Figure 2. Example of game where LXMERT correctly guesses the object, but RoBERTa does not.

As highlighted in the paper, it seems that the mistakes done by RoBERTa are more human-like. An example pointing out this phenomenon is shown in figure 3, where models and one human receiving the whole dialogue except for the last turn selects the wrong target in the image. Reading the dialogue, it is clear that the target is not on the bike on the right side of the image. Nevertheless, LXMERT wrongly guesses the person on that bike. Roberta and one human, instead, guess the person standing on the left part of the image as the target, but they are wrong since the dialogue specifies that the target is on the bike.

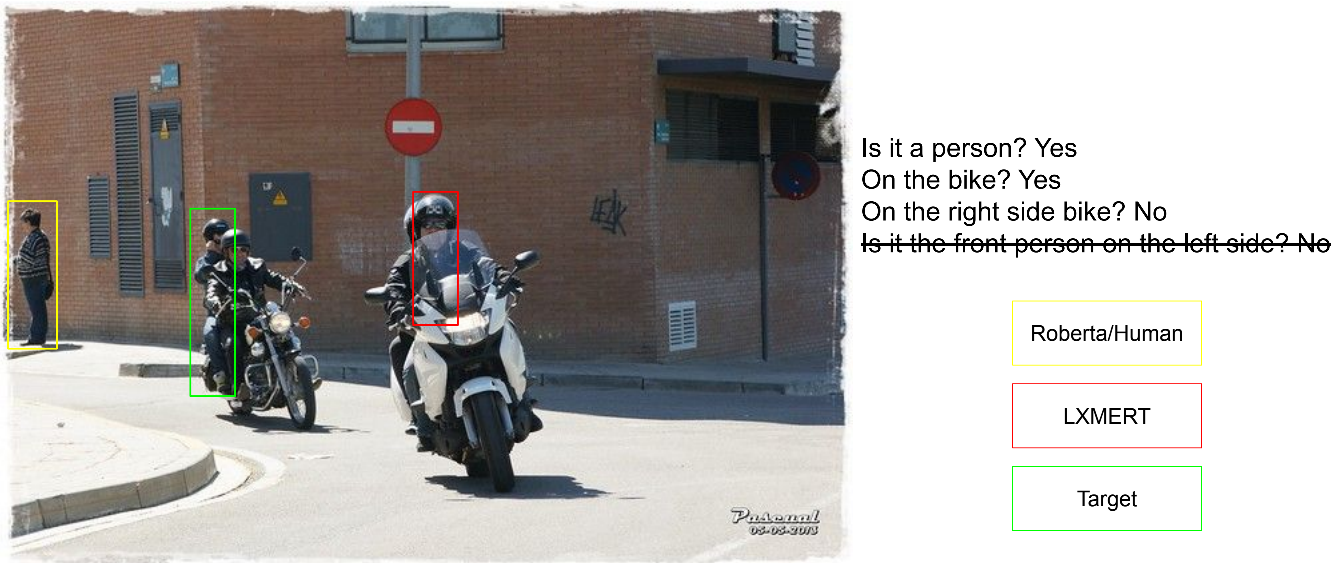

Figure 3. Example of game where models and humans are all wrong, but RoBERTa does the same mistake performed by one human.
